# Pathology as a phenomenological tool

**DOI:** 10.1007/s11007-021-09538-9

**Published:** 2021-03-09

**Authors:** Havi Carel

**Affiliations:** Department of Philosophy, University of Bristol, Cotham House, Bristol, BS6 6JL UK

**Keywords:** Phenomenology, Illness, Pathology, Husserl, *Epoché*

## Abstract

The phenomenological method (or rather, methods) has been fruitfully used to study the experience of illness in recent years. However, the role of illness is not merely that of a passive object for phenomenological scrutiny. I propose that illness, and pathology more generally, can be developed into a phenomenological method in their own right. I claim that studying cases of pathology, breakdown, and illness offer illumination not only of these experiences, but also of normal function and the tacit background that underpins it. In particular, I claim that the study of embodiment can be greatly enhanced, and indeed would be incomplete, without attending to bodily breakdown and what I term bodily doubt. I offer an analogy between illness and Husserl’s *epoché*, suggesting that both are a source of distancing, and therefore motivate a reflective stance.

## Introduction

Illness is a profound and life-changing event. Falling ill or receiving a diagnosis of a serious illness requires the ill person, and those around her, to stop and take stock of life as it has been and as it may be from now on.^1^ This often leads to a reconsideration of values and goals, and to a need to renegotiate ways of being that previously characterised the ill person’s life. Given the profound impact illness has on one’s life and its near-universal nature (we all grow old and die, most often from a disease of some sort), illness plays a significant role in human life. Although illness is a largely negative and painful experience, it is useful to bear in mind that it can also be edifying and the source of personal growth, in ways documented in philosophical, psychological and health economics literature.^2^ For these reasons, philosophy ought to study illness, but also to view illness as a philosophical resource, as I argued previously.^3^ The relationship between illness and philosophy is bilateral: philosophy is necessary for a full understanding of illness and the study of illness is integral to a philosophical investigation of human existence.

On a practical level, illness brings about change and demands adaptation. The ill person may be unable to continue working or may need help with daily care, for example. This requires readjustment and consideration where once routine and habit were sufficient. But there is also considerable change on an existential level, where illness is often experienced as a call for reflection. I suggest that illness is not only a life event that can be studied by philosophy, but also a *forceful invitation* to philosophise. I use the almost contradictory term “forceful invitation” to express its ambivalence. One the one hand, it is an urgent call, a demand for reflection. On the other hand, it remains optional. Not all who are invited will, or ought to, respond reflectively to illness. I suggest that illness plays an active role as a motivation for, or entry gate into, philosophy. The role of illness is not limited to that of a passive object of phenomenological scrutiny. Rather, I propose that illness, and pathology more generally, can be developed into a phenomenological method in their own right.

I further propose that the process a person undergoes when they fall ill is, in important respects, similar to Husserl’s *epoché*, or bracketing of the natural attitude. The two share important features: They both distance a person from habitual ways of being; they both offer open, non-prescriptive ways of experiencing; and they both present an alternative to a set of metaphysical assumptions we tacitly make about the world.

I claim that studying cases of pathology, breakdown, and illness illuminates not only these pathological experiences, but also normal function and the tacit background that underpins it. In particular, I claim that the study of embodiment can be greatly enhanced, and indeed would be incomplete, without examining bodily breakdown and what I term “bodily doubt.” Thus, in these two ways—the *distancing* that illness causes and the *insights* we may glean from illness experiences—illness offers a philosophical method proper.

In what follows, I explain the motivation for this view and outline what a “pathological phenomenology” might be like. I suggest that the study of pathology, whether of body or of mind, can form the basis of a phenomenological method. This is for several reasons. First, illness is a “*limit case,*” helping to reveal the full spectrum of human experience, because it adds extreme or abnormal experiences to what we normally experience. Second, illness often gives rise to *reflection* on finitude, dis-ability, suffering, injustice, and other existential themes. It tests conventional views, as well as norms and habits that have tacitly and pre-reflectively guided us. Third, illness *distances* the ill person from previous bodily and life habits and their accompanying tacit assumptions. This gives me reason to suggest that illness amounts to an entry gate into philosophical reflection and offers a phenomenological method in its own right.

A final note on my use of the term pathology: I loosely follow George Canguilhem’s distinction in *The Normal and the Pathological*, between normal states that are conducive, “business as usual” states of the organism, which express a harmonious relationship with the environment, and pathological states, which are states of difficulty, aberration, and disharmony.^4^ I am not suggesting a normative hierarchy privileging the normal over the pathological *tout court*; no such simple hierarchy is possible and the value judgements we make about these states are complex and context dependent. What I do argue is that there is a profound break between the two types of state so pathological states are not merely amplified or quantitatively more extreme versions of normal states, but of a different kind altogether and hence worthy of independent and thorough philosophical investigation.

## Illness as existential change

Illness is a disruptive, painful, frightening experience.^5^ It often shakes one’s being to the core, demanding an immediate response from the ill person and those around her. It is a powerful way to reveal the limits of embodied experience, considering the extraordinary bodily experiences that illness affords us, for example, when undergoing major surgery, suffering a stroke or cardiac arrest, and of course when dying. These experiences may bring the person suffering from them close to the limit of human existence and are can thus be called “limit cases”; such experiences often exceed our capacities to make sense of them.

These kinds of cases and other experiences of extreme and unusual embodiment shed light on normal experience, putting it in a new perspective and opening new horizons of bodily experiences as we know them.^6^ Illness has a powerful capacity to shed light on normal experience because it both extends it and contrasts with it. It broadens both the bodily and mental spectrum of experience, to include the pathological domain. For example, bodily experiences of sudden paralysis, debilitating pain, or losing a sense are all pathological experiences that necessarily broaden the spectrum of bodily experience because they are profoundly different to normal experiences and to the experiences which preceded them. The spectrum of mental experiences can also be broadened through pathological experiences, for example, with the appearance of voice hearing or visual hallucinations.

Although we usually call illness experiences “pathological” to indicate that they lie outside the normal range of experience and are *harmful* (as opposed to merely neutral) dysfunction, they are still an integral part of life, as illness remains a near-universal event in human life, and thus part of what I have called, with Ian James Kidd, “the facts of life.”^7^ No account of human life, and certainly no account of the good human life, is complete without considering illness and pathology as currently necessary aspects of human existence.^8^

In terms of their contrast with normal experience, pathological cases are ones in which illness disrupts normal, previously taken for granted, transparent experiences of health, and replaces a past set of habits and routines with a newly acquired set of responsive adaptations to the illness. Illness can become salient in two ways: it can be experienced as a sudden and severe disruption (e.g. in the case of stroke), or it can gradually become a form of life (e.g. with the progressive decline of function characteristic of some illnesses).^9^ The important common feature of both processes is the disruption of past experiences and habits. In this illness differs from congenital disability, for example, where there is no disruption of this sort and hence little need to adapt, reorient or restore a disrupted life narrative.

But the two processes of becoming ill differ in how the disruption is experienced. In the first case, that of sudden illness or accident, the disruption is foreign, threatening, and sudden. It is an abrupt break from previous events which stands in sharp contrast to them and thus makes visible the previously largely transparent structure of everyday life. In the second type of experience, illness becomes a form of life. It “sneaks up on us, or we become so habituated to it that it defines our form of life—it becomes us, or we become it.”^10^ As “it becomes us,” our old life form is shed and a new form—with illness—becomes the “new normal.” There is a difference from past everyday experience, but this change has come about gradually. This is the case of, for example, a slow progressive disease that gradually restricts the ill person’s world. Gradual accommodation may mean that the illness is not experienced as a dramatic disruption but as a gradual change. These could also be two phases of an illness, which may at one time progress slowly and almost imperceptibly and at other times cause sudden exacerbation.^11^

Looking more closely at this view of illness as a “complete form of existence,” we can see how a new lifeworld, a restricted, dis-eased one, can replace the previous lifeworld of health, bodily transparency and “silence of the organs.”^12^ What makes it a new lifeworld are not only practical concerns, but the fundamental fact that *all* of one’s modes of being and doing are affected by illness, for example, by changes to motility, energy levels, pain and sensory experience, as well as to mood, social world, goals and projects, relationships with others, temporality and so on. Illness profoundly alters how we find ourselves in the world, radically modifying the embodied basis of the *existentiales* (in the Heideggerian sense) upon which meaning, sociality, identity, selfhood, autonomy, and consciousness are premised.^13^ The foundation upon which a life is built is fractured by illness, and is then sometimes replaced by a new foundation from which novel modes of being can be rebuilt, but this foundaion can also be destroyed with nothing to replace it.

To illustrate the profound change to the ill person’s world, consider our concepts. One’s concepts change with illness. If one now needs to use a wheelchair, the upper floors of a building with no lift become inaccessible. But beyond the practical problem now faced by the person, shared meanings (such as “the common room on the top floor”) become disrupted. Terms such as a “short walk” or “round the corner” lose their shared meaning and acquire new, idiosyncratic meaning by the ill person, who has now moved away from shared meaning not only practically and socially but also conceptually: the norms which underpin much human exchange no longer include her embodied being in the world.

The ill person’s engagement with the ready-to-hand environment in which they find themselves also changes. As Kay Toombs writes, “the bookcase outside my bedroom was once intended by my body as a ‘repository for books’ then as ‘that which is to be grasped for support on the way to the bathroom,’ and is now intended as ‘an obstacle to get around with my wheelchair’.”^14^ The relationship the ill person has with objects, people, space and time, shifts as the illness progresses.

In response, the ill person’s expectations also change. She may expect to do less, give up certain activities (e.g. travel, sports) and realign her plans, values and ambitions accordingly. For example, a healthy person would probably not be satisfied by a prognosis of 5-year survival, whereas someone with an aggressive cancer may consider that an enviable goal. The ill person’s sense of time and value are often readjusted. Illness is a comprehensive realignment of meaning, values and ways of being.^15^

This degree of disruption, adjustment or adaptation calls upon the ill person to engage reflectively with their life and open themselves to the existential challenge of diminished bodily capacity, mental incapacitation, pain, and fear of the future.^16^ The engagement can be solely on a practical level, but some illness events, such as receiving a poor prognosis, inevitably raise questions about death, meaning, and value. These kinds of questions trigger reflection, for example through deep conversations with friends, carers or family.^17^ Thus, illness *can* be—although, importantly, this is not a prescriptive statement—an invitation to engage critically with existential questions,^18^ as Arthur Frank writes:

Critical illness offers the experience of being taken to the threshold of life, from which you can see where your life could end. From that vantage point you are both forced and allowed to think in new ways about the value of your life. Alive, but detached from everyday living, you can finally stop to consider why you live as you have […].^19^

The disruptions and existential demands of illness amount to a powerful *distancing* of the ill person from her now-lost healthy life. This distancing can motivate a reflective stance and is a forceful invitation to philosophise. As such, the distancing triggering this reflective process is a type of what Husserl calls the *epoché*, to which we now turn.

## Illness as epoché

Illness, I suggest, is an existential change of such magnitude that it amounts to a kind of *epoché*, or bracketing, putting in abeyance our former beliefs about the nature of reality. The reflective process triggered by illness can also be thought of as a form of phenomenological reduction. The reduction is core to phenomenological practice and involves moving our attention from objects to acts of perception, focusing on the acts, their meaning, modes of operation and how we experience them. Importantly, the reduction does not involve ceasing to perceive, nor is it a sceptical stance.^20^ Rather, it is a shift in a way of being that suspends our natural attitude towards perception and the world and enables philosophical reflection. The reduction calls on us to suspend our underlying metaphysical beliefs, in favour of a neutralization of belief in the existence of the world or of a particular object. The reduction brackets our realist assumptions to enable withdrawal from an ordinarily implicit commitment to the reality of the world. As Zahavi notes, the reduction’s role is not to “deny, doubt, neglect, abandon, or exclude reality from our research, but simply to suspend or neutralise a certain dogmatic attitude toward reality.”^21^ In the reduction, under-theorised aspects of experience become an object of enquiry, because we shift our attention from a given object to the way in which it is given and its modes of appearance to us.

For Husserl, this procedure enables us to bracket the natural attitude, and thus to set aside, or suspend, taken-for-granted, meaning-laden, metaphysically determined ways of experiencing. Phenomenology is committed to making explicit aspects of experience that are overlooked by other approaches and may be poorly understood. An adequate approach to the experience of illness requires a phenomenological reduction: a suspension of a natural attitude of implicitly accepting the background sense of belonging to a world and various interpretive dogmas along with it. Bracketing the natural attitude is a withdrawal from the ordinarily implicit commitment to the reality of the world, which allows us to see the world as a phenomenon of being, instead of something that is.^22^

In order to reflect in this way, one needs to dislodge everyday habits and familiar modes of experience. Again, this is not a removal from the world but a shift to a way of being in the world that enables reflection. The shift moves us from the natural to the critical attitude, in which one can examine their relationship to the world in acts of perception in a new light. As Husserl writes, this “Inhibiting” or “putting out of play” of the natural attitude exposes “my pure living [...] the universe of phenomena.”^23^ Zahavi characterises the reduction as an “entry gate” to reflection.^24^ In the next section I suggest that the reduction, and illness, are such an entry gate to reflection. But first, I would like to draw out the commonalities between the reduction and the distancing caused by illness.

There are several ways of thinking about the relationship between the *epoché* and illness. We should consider disability, as well as the two forms of illness, sudden and gradual, discussed earlier. We also need to distinguish congenital from acquired disability. There may be considerable overlap between the different categories, so it is worth reminding the reader that I use the term illness here to denote a serious health condition that may be chronic and is not fully reversible. There is much to say about the different categories and how this definition would relate to each, but it will suffice to suggest a rough definition: I wish to pick out the group of conditions that amount to a serious and irreversible health condition, which may be caused by disease but may also be caused by trauma, e.g. an accident. The irreversibility is important, as full recovery enables a return, so to speak, to the natural attitude, whilst remaining ill requires developing an alternative attitude, as a return to the pre-reflective attitude is not possible.

Illness is bracketed by refusing to see it solely as a physiological process, the object of biomedical enquiry.^25^ The shift is to viewing illness as an experience, which may or may not be linked to physiological dysfunction (for example, in some cases of mental illness, such as personality disorders there is no such known link). When we bracket in this way, we resist the common contemporary (Western) understanding that the disease (biological dysfunction) is the correct object of investigation, and instead turn our attention to the illness (the experience of feeling “dis-ease”). This enables us to decouple the illness experience from a biomedical framework, social scripts and the “sick role,” which are common ways in which illness is reduced to disease while alternative interpretations of it may be suppressed to the point that they are eradicated.^26^

Resisting reductive dominant perspectives, which posit the illness experience as derivative from other, more primary, entities or processes, is an important kind of resistance phenomenology offers. Resisting the impulse to categorise, define, and reduce experience is a major task of phenomenology. In the case of illness, phenomenology can serve as an antidote for some kinds of hermeneutical injustice, a concept coined by Miranda Fricker.^27^ Hermeneutical injustice is one kind of epistemic injustice, which is caused by epistemic marginalisation in which only the dominant interpretation of the experience, in this case the biomedical view, is accepted as accurate, true, and fully representative of the reality of illness.^28^ Hermeneutical injustice arises from a lack in interpretative resources that would enable an alternative understanding of an experience, in this case the experience of illness. As long as patients simply mimic the medical jargon and manner of speaking about their illness, no alternative interpretation can emerge. But if ill persons insist on developing their own interpretation and resisting the pre-determined given ones (e.g. resisting the biomedical view of one’s illness as reducible to disease), it can be experienced and articulated idiosyncratically, personally, subjectively, and non-reductively.^29^

The phenomenological patient toolkit provides patients with tools for cultivating alternative, non-dominant interpretations of their illness experience.^30^ It is also designed to help patients move away from the natural attitude towards illness. The toolkit provides a non-judgemental, supportive, and open context in which its three steps can help ill persons make their own sense of their experience of illness, using phenomenology. Bracketing the natural attitude toward illness suspends the belief in the reality of an objective disease entity. This suspension does not deny the objective reality of disease processes; but shifting the focus away from the disease entity and toward one’s own experience of it can disclose new features of this experience, as well as giving ownership over the illness experience and the ensuing process of sense-making.

We usually take the disease entity for granted and posit it as the source of the illness experience. But, in fact, for the ill person the illness experience often comes *before* (both temporally and in terms of its importance) the objective disease entity and in some cases (e.g. medically unexplained symptoms, some mental disorder) is never anchored in any known disease entity.^31^ Once the belief in the objective disease entity is bracketed and we are distanced from our usual way of experiencing, we can begin to explore how illness appears to the ill person, and what are its structure and features.

This bracketing is the first step in the phenomenological toolkit. The toolkit uses bracketing to develop a new understanding of an illness phenomenon and how it can help resist pre-given interpretations of the illness experience. This bracketing of the natural attitude is analogous to Husserl’s *epoché*, so illness both triggers the *epoché* but can also be interpreted by it.

I suggest that illness forces a kind of *epoché* on the ill person. What one ordinarily takes for granted becomes salient when it is lost or changed through illness. Illness involves a phenomenological reduction because it compels us to suspend “our normal, taken for granted way of approaching the world.”^32^ This is particularly so in the case of mental illness, which involves changes to the natural attitude and therefore requires a methodological shift to understand it.^33^ These phenomenological insights can help ill persons engage in an unrestricted fashion with their illness. The *epoché* can pave the way to exploring the unique significance illness has for a particular individual, within a particular context and situation.

The second step in the toolkit is thematising illness. “Thematising” refers to the act of attending to a phenomenon, which makes aspects of it explicit.^34^ A theme for a particular consciousness is that upon which it focuses its attention. This does not simply denote the intentional object; it also takes into account the kind of attentional focus on an entity. Thematising may include attending to the cognitive, emotive, moral, or aesthetic aspects of a phenomenon. A patient may thematise her illness as a central feature of her life, attending to her symptoms as pervasive, while the physician may thematise the illness as a “case of cancer,” attending to symptoms as diagnostic clues.^35^ The understanding that illness is not an objective entity and the exercise of thematizing may help patients because it enables moving away from prescriptive pronouncements toward a descriptive mode.

Thematising can be used for bringing out the multiple perspectives on one’s illness that patient, family, health professionals, and others may have, as each will thematise an illness differently. The patient may thematise her illness emotively, while a health professional will thematise it cognitively. A family member may thematize illness as an experience of empathy. Thematising is useful for uncovering the variety of ways of appearing illness has. Exploring the different thematic centres illness may have can illuminate its multiple ways of appearing. By thematising different aspects of illness as a social phenomenon, as a source of pain, as a trigger of innovation, and so on, the multidimensionality of illness can be revealed. Thematising creates a complex, shifting, view of illness as moving from foreground to background, as changing in meaning, and as consisting of multiple perspectives. Also, the theme of one’s particular concern is presented against a background, the horizons in which it appears.^36^ Understanding the figure–background relationship helps to see illness as part of a broader context, and illness horizons that are more productive can be explored.

The third step of the toolkit is to take the new understanding of illness (as a distancing that has been thematized) and examine how it changes one’s being-in-the-world. The main components of being-in-the-world, for Heidegger, are being-in (inhabiting or dwelling in a place), the world (the meaningful network of entities, practices, and meanings that make up our world), and being, which is the open existence of humans, capable of temporal existence and understanding.^37^ The toolkit uses being-in-the-world to capture the pervasive effects illness may have on one’s sense of place, interactions with the environment and with other people, on meanings and norms, and on the nexus of entities, habits, knowledge, and other people that makes up one’s world. This term enables us to elaborate on the impact of illness richly and comprehensively. By moving away from a narrow understanding of illness as a biological process, ill persons can develop a thick account of illness as a new way of being-in-the-world. Because illness turns from being an external intrusion to being a form of existence, the notion of being in the world is particularly apposite; it helps us understand the pervasive impact illness may have on all life domains, which are interconnected, as captured by the term “being in the world.”

The patient toolkit is a practical exercise of the *epoché*, and a theoretical means of demonstrating the analogy between illness and the *epoché.* The reduction, which is a key tool in phenomenological work, as well as other tools, such as thematising, are not just useful tools for illuminating the experience of illness. This illumination is also a form of resistance, eschewing external understandings of illness foisted upon the ill person. The toolkit opens a space within which an idiosyncratic and individual interpretation can be developed independently of any pre-given interpretations, such as social scripts and biomedical interpretations. Such resistance is important not just politically, in terms of patient autonomy and patient rights, for example, but also philosophically, because it demonstrates the power of illness to generate reflection. In this illness is a sister process to the *epoché.* The dual role of illness, as philosophical trigger and as prime candidate for philosophical attention, is also evidenced in the kind of personal crisis that often accompanies falling ill, to which I now turn.

## Illness, crisis of meaning, and reflection

The process described in the previous section affirms the analogy between illness and the *epoché* as both powerful and useful. Illness, like the *epoché*, strips away shared meanings (bracketing, or the first step of the toolkit), and in doing that sheds new light on ordinarily tacit aspects of existence. I now want to continue developing this account by looking at illness as a crisis of meaning. Illness can be a life crisis, especially if the diagnosis or symptom appearance are sudden. It is characterised by disruption, detachment from the everyday, and can amount to a collapse of the ill person’s narrative. This collapse can initiate deep anxiety, which Heidegger characterises as a loss of meaning, in which possibilities for action become levelled as they lose their salience and make one unable to choose between them. One can then fall into a state of paralysis, unable to act, because unable to choose a course of action, as all options have been levelled. In this state action is paralysed but existence is experienced at its most acute, as Heidegger describes in *Being and Time*.^38^ Illness can operate similarly to Heidegger’s state of anxiety, paralysing the structures and habits of everyday life that served until now to scaffold one’s actions. Both Husserl’s *epoché* and Heideggerian anxiety are stances in which significant experiences give rise to reflection. I suggest that the experiences illness, the *epoché*, and anxiety share are all reflective, or even hyper-reflective, forms of engagement with the world.

The case of illness is particularly interesting because reflection is a demand placed upon the ill person, who is compelled to re-examine and change her life. Illness is a violent event, an encounter which destabilises the structures of experience. Illness reveals our being by pushing it to its limits, to finitude, dis-ability, and alienation which are extreme modes of being. Illness may be even more radical than the *epoché* because it is imposed upon the ill person with no invitation, often little preparation, and little to protect oneself from its impact.

I now want to offer a view of illness that goes beyond this catastrophic and destructive picture, and suggest that, without denying the hardship and adversity of illness, it can also be a way into philosophy. Because it is a radical displacement of the natural attitude which problematises one’s relationship to one’s world, illness can lead to critical philosophical reflection. It does not necessarily do so and indeed, people who are ill may be too engaged with their personal struggle to want or be able to reflect. But illness gives rise to disruption, change and challenge in ways that amount to an existential jolt that may lead people to philosophise.

For example, illness is often said to lead to objectification. In this process, there is a growing distance between self and body, which is reified by medical inquiry and treatment. Illness breaks down the natural attitude towards the now-ill body and disrupts the unity between the body as object and the body as subject, as well as making objectification replace the habitual body. Jean-Dominique Bauby writes in his moving account of his stroke which left him with locked-in syndrome, *The Diving Bell and the Butterfly*:

Reflected in the glass I saw the head of a man who seemed to have emerged from a vat of formaldehyde. His mouth was twisted, his nose damaged, his hair tousled, his gaze full of fear. One eye was sewn shut, the other goggled like that doomed eye of Cain. For a moment I stared at that dilated pupil before I realised it was only mine.^39^

Illness can often also lead to a feeling of uncanniness. The ill body is a source of negative experiences, so that the body becomes an obstacle and threat. The perspicuous nature of bodily orientation becomes occluded with negative attention. The Husserlian attitude of practical ability, the sense of “I can” is replaced by “I cannot,” an attitude of helplessness, resignation, dis-ability. And these experiences can lead to what Fredrik Svenaeus calls “unhomelike being in the world.”^40^ It is not just new experiences that give rise to these feelings of objectification and uncanniness. Illness can, more radically, change the structure of experience by modifying the experience of space and time, as we see happening to time consciousness in depression.^41^ Objectification and uncanniness can also invite reflection, because they are so different to previous experience: they consist of a rift from past ways of being embodied.

Illness is an invitation to a unique form of reflection. Whereas we usually think of reflection as volitional and theoretical, in this case it arrives through illness, uninvited and threatening, causing anxiety and uncertainty. Illness is a radical, violent philosophical motivation which may also be more radical, more intimate, and more authentic than theoretical entry gates, such as the *epoché*. It is more intimate because it ensues from one’s experiences of one’s own body or mind changing abruptly and radically. As such it calls for a more radical or personal method of philosophising, through a phenomenological method. The radical nature of this invitation can also be used to sharpen and expand philosophical enquiry. Illness as philosophical invitation can affect the ill person’s philosophical concerns, as well as lending urgency and salience to the philosophising it motivates.

## Pathology illuminating normalcy

Illness deepens our understanding of embodied living by illuminating normal patterns of motility, comportment, and spatiality through their aberration. Merleau-Ponty uses this methodology when he examines pathological cases, such as phantom limb, anosognosia, and aphasia, to illuminate normalcy as full and spontaneous engagement with the world.^42^ This methodology has been criticized as creating a false dichotomy between normalcy and pathology and as conflating natural and normal function.^43^ However, it is undeniable that illness raises philosophical questions about embodied existence, the mind–body relationship, value and meaning of life, death and finitude, and human vulnerability. As a juncture of such central philosophical issues, it deserves systematic philosophical exploration not just of these issues and themes, but also of its process, structure and edifying capacity.^44^ Illness is a pragmatic disruption of lived experience, not an academic, studied phenomenological reduction but a lived experience that motivates such a reduction. As such it is phenomenology in action. However, the contribution illness can make to phenomenology, and philosophy more generally, has been overlooked.

Pathological states of embodiment enable us to see the full range of human experience (as discussed in Sect. 2) as well as the contrast between normal embodied experience and its aberration.^45^ Pathological instances shed light on normalcy by contrasting with taken-for-granted normal situations, perceptual and motor processes, and modes of being. This accords such states a productive epistemic role in, for example, phenomenology, philosophy of perception, epistemology and philosophy of action. The reliance of much philosophical argument on normal embodiment, which is a common tacit assumption in many contemporary discussions, can lead to a narrowing of possibilities of thought and a restricted consideration of what human life is and can be. Looking to expand this menu is an important task, one that only now is beginning to be undertaken by a variety of authors.^46^

Cases of pathology, e.g. neuropathology, demonstrate to us that normal perception is not something taken for granted but an *achievement*. Merleau-Ponty’s well-known analysis of Schneider, for example, illustrates that. Merleau-Ponty characterises Schneider’s malaise as *existential*, as modifying his entire existence. “[Schneider] is ‘bound’ to the actual, and he ‘lacks freedom,’ he lacks the concrete freedom that consists in the general power of placing oneself in a situation.”^47^

Merleau-Ponty uses Schneider’s peculiar pathology to reveal how our bodies are “the power for a certain world.”^48^ The normal person, Merleau-Ponty says, “reckons with the possible,” which thus acquires a certain actuality.^49^ In pathological cases the field of actuality is limited—much of what was possible is now off-limits. What was effortless and taken for granted is now a conscious, explicit effort; it is this effort and the correlating achievement of action that make pathological cases illuminating, because this effort illuminates for the first time taken for granted ways of being.^50^ By making explicit what normally goes unnoticed, such cases draw our attention to how things normally are.

Merleau-Ponty’s interpretation of Schneider’s case can be formulated in a more general form as the breakdown of the intentional arc:

[…] the life of consciousness – epistemic life, the life of desire, or perceptual life – is underpinned by an “intentional arc” that projects around us our past, our future, our human milieu, our physical situation, our ideological situation, and our moral situation […]. This intentional arc creates the unity of the senses, the unity of the senses with intelligence, and the unity of sensitivity and motricity. And this is what “goes limp” in the disorder.^51^

This breakdown of normal human existence, which can happen in a vast range of somatic and mental disorders, merits philosophical study in its own right.^52^ But it also provides a unique opportunity to reveal facets of normal existence that are usually invisible. Pathological cases can make explicit the intricate life of embodied consciousness which gives rise to everyday experience.^53^

I now turn to a somatic case which will be less familiar. This example is the study of pathological breathlessness, undertaken in the *Life of Breath* project, a research project led by the author and Jane Macnaughton.^54^ Studying breathlessness that is caused by clinically subnormal respiratory or cardiac function can reveal tacit aspects of the normal sensation of breathlessness, experienced when healthy people exercise, for example.^55^ Pathological breathlessness reveals the extent to which natural breath modulation supports everyday action, how the brain produces the sensation of breathlessness using environmental cues and past experiences, and how restricting breath has an immediate and powerful impact on one’s emotional state.^56^

Empirical studies of the sensation of breathlessness reported by respiratory patients describe it as threatening, limiting, and unpleasant, giving rise to a feeling of loss of control and to profound suffering.^57^ This can make explicit the feelings associated with normal breathlessness experienced by healthy people on exertion, e.g. when dancing or playing a sport. Normal breathlessness contrasts sharply with pathological breathlessness: it is benign, can be enjoyable, and is not threatening or uncontrolled. It goes largely unnoticed and therefore does not call upon the person experiencing it to reflect, whereas pathological breathlessness does.

Other dimensions of pathological breathlessness also illuminate normal breathing. Panic attacks, in which people feel unable to breathe, or feel they are suffocating, reveal the normal sensation of bodily certainty that underpins each breath. Doubting our breath, or any other bodily function, unravels our bodily certainty, resulting in loss of continuity, loss of transparency of the body, and loss of faith in one’s body.^58^

Looking at this stanza by Rilke, we can see how significant, yet tacit, rhythmic breathing is to our experience:

Breath, you invisible poem---

pure exchange, sister to silence,

being and its counterbalance,

rhythm wherein I become.^59^

Breath is invisible, and akin to silence, but is nonetheless the source of the balance and rhythm of becoming. It is only when breath is disrupted that the normal way of things—the rhythm it provides, the “pure exchange” it continually facilitates—comes to our notice. Breathing and its pathological counterpart, breathlessness, are mutually implicated and mutually illuminating. Herein lies the usefulness of pathology: we need to study the pathological in order to bring to light normal and hence largely overlooked experiences. To understand what a certain brain area does, we study stroke victims with damage in the relevant area. To understand the importance of early years nurturing, we study its deprivation. To study breath, we look at breathlessness. In this way, the pathological can illuminate the normal, by making the invisible visible.

Merleau-Ponty writes: “[reflection] loosens the intentional threads that connect us to the world in order to make them appear; it alone is conscious of the world because it reveals the world as strange and paradoxical.”^60^ With these examples in mind, I suggest that illness, and pathology more generally, enact such a loosening of the intentional threads. Such enactment makes evident one’s complex, yet seemingly unremarkable—and therefore unremarked upon—relationship to the world.

I end on a speculative note. Might there be a useful analogy between cases of bodily or somatic pathology, such as the ones described above, and philosophical thought experiments? Are thought experiments examples of pathology within philosophy, and do they thus illuminate the normal form arguments and intuitions take? Can we think of established thought experiments—brain in a vat, the land of fake barns, some forms of scepticism—as ones that similarly distance us from habitual modes of reflection, away from the natural attitude, in favour of a more critical engagement with ideas that remove them from their sedimented, already-given meaning? Such dislodging, disruption and distancing are core to philosophical work and, I suggest, share the very same distancing force that is at work in illness and the *epoché*. The study of pathology in this wider sense is a new and *prima facie* merited form of phenomenological work, which I hope to further develop into a fuller form in future work.

## Footnotes

1 I use the term “illness” throughout to denote serious, chronic, or life-changing illness. I exclude from this definition transient illness (e.g. flu), or a medical condition that can be fully resolved (e.g. fractured bone in a healthy person).

2 Kidd (2012) and Haidt (2006).

3 Carel (2014, 2016b).

4 Canguilhem (1991).

5 Sontag (1978) and Carel (2018).

6 Carel (2013, 2016b).

7 Carel and Kidd (2019). On harmful dysfunction see Wakefield (1992).

8 cf. Carel (2016b, ch. 6).

9 Gallagher (unpublished presentation).

10 (ibid).

11 Paterson (2001).

12 Leriche, cited in Canguilhem (1991). On the transparency of health see Sartre (2003).

13 Heidegger (1962).

14 Toombs (1995, p. 16).

15 Carel (2016b, 2018).

16 Carel (2018).

17 See, for example, Albom (2003), Ware (2012), and Kalanithi (2016).

18 In addition, illness can also be an opportunity, indeed a demand, to demonstrate virtues such as courage, equanimity and patience. See Carel (2017), Kidd (2012).

19 Frank (1991, p. 1).

20 Zahavi (2003).

21 Zahavi (2003).

22 Ratcliffe (2008, p. 4).

23 Husserl (1960, p. 20).

24 Zahavi (2003), cf. Carel (2014).

25 Carel (2012).

26 Kidd and Carel (2018).

27 Fricker (2007).

28 Kidd and Carel (2016a, b, 2018).

29 See Kidd and Carel (2018) for an analysis of the epistemic injustice caused by naturalism in health care and medicine.

30 Carel (2012).

31 Toombs (1987).

32 Moran (2000, p. 144).

33 Ratcliffe (2008, p. 7).

34 Toombs (1987, p. 222).

35 (ibid.).

36 Drummond (2007, p. 201).

37 Heidegger (1962).

38 Heidegger (1962).

39 Bauby (2007, pp. 32–33).

40 Svenaeus (2000a, b).

41 Ratcliffe (2012).

42 Merleau-Ponty (1964, 2012).

43 Dorfman (2005).

44 cf. Kidd (2012).

45 Carel (2016b).

46 See, for example, Harcourt’s (2016) and Carel’s (2016a, 2017) attempt to do this for virtues; Barnes (2016) bringing the minority body into philosophical mainstream; Stone’s (2019) attention to natality; Leder’s focus on the distressed body (2016); and Slatman’s (2014) *Our Strange Body*.

47 Merleau-Ponty (2012, p. 137).

48 (ibid., p. 109). It was thought that Schneider’s pathology was vision agnosia, but recently Marotta and Behrmann (2004) have argued that his was a case of integrative agnosia.

49 (ibid., p. 112).

50 Gallagher (2005).

51 Merleau-Ponty (2012, p. 137).

52 Carel (2016a, b).

53 Another well-known case is that of Ian Waterman, who suffered from de-efferentation (loss of the motor nerve fibres) from the neck down (Gallagher 2005). Waterman learned to use vision to locate his limbs and identify his posture. Gallagher uses this case to provide a philosophical account of proprioception.

54 The *Life of Breath* project (2014–2020), funded by the Wellcome Trust, studied the experience of breathing and breathlessness. See www.lifeofbreath.org.

55 Carel, Macnaughton and Dodd (2015).

56 (ibid.), Faull et al. (2017), and Williams and Carel (2017).

57 Abernethy and Wheeler (2008).

58 Carel (2013).

59 Rainer Maria Rilke, *Sonnets to Orpheus*, Book II.

60 Merleau-Ponty (2012, p. xxvii).
